# Rural Water Supply

**Published:** 1905-09-09

**Authors:** 


### Rural Water Supply.

An interesting paper on Rural Water Supply, by Professor Sims Woodhead, which appears in the September issue of the "Journal of the Royal Sanitary Institute," calls attention to a somewhat neglected aspect of a very important national question. Indications have not been wanting, of late years, of a tendency on the part of wealthy municipalities to obtain water for themselves at whatever cost, and from very distant sources of supply; and it seems to be by no means a visionary danger that great works executed for this purpose may unfairly withdraw water from localities very ill able to part with it, and may entail, especially after prolonged periods of drought, very serious consequences upon rural populations. Even as matters now stand, the supply of a rural district is often exceedingly uncertain in quantity, and is seldom trustworthy in point of quality. Many farmhouses and hamlets are entirely dependent upon shallow wells sunk in porous soils, and liable to surface pollutions often of an extremely dangerous character; while it is by no means uncommon, after a dry summer, for even this supply to fail. In such circumstances a compulsory absence of cleanliness is, perhaps, the least of the evils certain to arise, and the village or villages concerned are only too likely to repeat the experience of the times before Dr. William Budd, when such places were the favourite seats of enteric fever, and the chief sources from which it spread to other and more favoured localities.

The new Metropolitan Water Board has already put forth the suggestion that it ought to be converted into a national one, in constant touch with local or county branches or committees, and empowered, with their assistance, to deal with the water supplies of England and Wales upon some uniform and carefully considered system, directed at once to the protection of water from excremental pollution, and to the establishment, in every locality, of a protected supply adapted to its wants. As Professor Woodhead points out, every case of typhoid fever is not only a source of direct expense to the locality in which it occurs, but is also a possible source of infection over far wider circles than is usually appreciated. An agricultural district far away from London may through its contaminated water supply become a centre of infection to the area in London in which milk from that district is distributed; and what applies in such an extreme case may apply still more directly to other agricultural areas and towns. The Professor declares that facts pointing in this direction were brought home to him very forcibly, on his being informed by a large London milk company that they had been unable to accept the milk from certain farms within a short distance of Cambridge, because they found that the water-supply of those farms was far from satisfactory. He assumed, not without reason, that, as the Londoners would not have the milk, the residents in Cambridge were compelled to consume it themselves, and with it any organisms and organic matter which might have been introduced with the water. The late Dr. Vivian Poore afforded experimental proof of the possibility of protecting surface wells effectually; and there is no doubt that much might be done in this direction, and with great public advantage, if those terrible persons, the so-called "local authorities," could only be aroused to some interest in their duties, and to some perception of the degree in which these duties are commonly neglected. The equalisation of supply would be a more serious matter; and for this, no doubt, some national, or at least county organisation is required. We are in complete theoretical agreement with the Metropolitan Water Board upon this part of the subject, but our short experience of the operations of that body has not yet been sufficient to justify any material extension of the powers at present placed in their hands. Their attitude, so far as we have had opportunities of observing it, has been somewhat that of a body of kindly disposed autocrats, who might easily be aroused to anger by any attempt to show that they themselves have duties, or that the ratepayers have rights. They are quite willing to supply water, so long as this can be done without inconvenience to their servants; but, when Mr. Bumble puts on his official costume, the public must be content to go to the wall.

